# Serum and exhaled breath condensate inflammatory cytokines in community-acquired pneumonia: a prospective cohort study

**DOI:** 10.1186/s41479-016-0009-7

**Published:** 2016-06-23

**Authors:** Stefano Aliberti, Letizia Corinna Morlacchi, Paola Faverio, Rafael Fernandez-Botran, Roberto Cosentini, Marco Mantero, Paula Peyrani, Julio Ramirez, Jose Bordon, Francesco Blasi

**Affiliations:** 1 Department of Pathophysiology and Transplantation, University of Milan, IRCCS Fondazione Ca’ Granda, Ospedale Maggiore Policlinico, Milan, Italy; 2 School of Medicine and Surgery, University of Milan Bicocca, Respiratory Unit, AO San Gerardo, Monza, Italy; 3 Department of Pathology and Laboratory Medicine, University of Louisville School of Medicine, Louisville, Kentucky USA; 4 Emergency Medicine Department, IRCCS Fondazione Ca’ Granda, Ospedale Maggiore Policlinico, Milan, Italy; 5 Division of Infectious Diseases, Department of Medicine, School of Medicine, University of Louisville, Louisville, Kentucky USA; 6 Department of Medicine, Section of Infectious Diseases, Providence Hospital, Georgetown University Medical Center, Washington, DC USA

**Keywords:** Inflammatory cytokines, Exhaled breath condensate, Community-acquired pneumonia, Serum, Inflammation

## Abstract

**Background:**

The role and relationship between pro- and anti-inflammatory cytokines represents one of the least studied aspects of the pathogenesis of community-acquired pneumonia (CAP). The aim of the present study was to evaluate pro- and anti-inflammatory cytokines at both local (lung) and systemic (blood) levels and their relationship with the severity of the disease on admission and time for a patient to reach clinical stability during hospitalisation.

**Methods:**

This was an observational, prospective, cohort study of hospitalised patients with a diagnosis of CAP at the IRCCS Policlinico Hospital, Milan, Italy, between April 2010 and January 2012. Ten pro-inflammatory cytokines (interleukin [IL]-1, IL-1α, IL-1β, IL-2, IL-6, IL-8, tumor necrosis factor [TNF]α and interferon [IFN]γ) and anti-inflammatory cytokines (IL-4 and IL-10) were measured in both serum and exhaled breath condensate within 24 h after hospital admission.

**Results:**

A total of 74 patients (median age: 76 years; gender: 61 % male) were enrolled. The anti- to pro-inflammatory cytokine ratio was reduced in patients with severe disease on admission and prolonged time to reach clinical stability. This was due to lower levels of anti-inflammatory cytokines in the exhaled breath condensate and higher levels of pro-inflammatory cytokines in serum.

**Conclusions:**

Dis-regulation between pro- and anti-inflammatory pathways might be a part of the pathogenic mechanisms that lead to severe infection and worse early clinical outcomes in CAP patients.

**Electronic supplementary material:**

The online version of this article (doi:10.1186/s41479-016-0009-7) contains supplementary material, which is available to authorized users.

## Background

Community-acquired pneumonia (CAP) still represents a leading cause of death among infectious diseases, with mortality rates that have not decreased since the introduction of antibiotics in the early 20th century [1–3]. Recently, special attention has been focused on the double role that inflammation plays in the pathogenesis of this disease. On one hand, inflammation is part of the host’s protective immune response against microorganisms; on the other hand, if excessive, inflammation can be detrimental and increases patients’ morbidity and mortality [4].

Previous studies taught us that, in patients with pneumonia, a rise in systemic pro-inflammatory cytokines worsens clinical outcomes, lengthens time to recovery, and increases levels of lung injury [5–8]. However, most of these data evaluated cytokines in serum, while the presence and characterisation of inflammation in the lung has not been fully explored. When inflammation was investigated in bronchoalveolar lavage (BAL) and blood of patients with severe CAP, an increase in cytokine levels was found to be variably associated with increased lung injury, multi-organ failure, and mortality [9, 10]. Since BAL is an invasive technique, exhaled breath condensate (EBC) has been recently used to evaluate non-invasive inflammatory patterns in some respiratory diseases [11, 12], not including pneumonia.

The relationship between pro- and anti-inflammatory cytokines in the pathogenesis of pneumonia is another little explored field. An imbalance between pro- and anti-inflammatory responses seems to be associated with respiratory failure and mortality in patients with *Pneumocystis jirovecii* pneumonia [13]. We hypothesise that an imbalanced anti- to pro-inflammatory cytokine ratio in patients with CAP on hospital admission might be related to a higher severity of the disease on admission and a prolonged time to reach clinical stability (TCS) during hospitalisation.

The aim of the present study was to evaluate: (i) the inflammatory pattern in both blood and EBC in CAP patients on hospital admission; (ii) the relationship between pro- and anti-inflammatory cytokines (separately in blood and EBC) and the severity of the disease in CAP patients on hospital admission; and (iii) the relationship between pro- and anti-inflammatory cytokines (separately in blood and EBC) and TCS in patients with CAP.

## Methods

### Study design, setting and patients

This was an observational, prospective, cohort study of consecutive patients admitted with a diagnosis of CAP at the pulmonary and emergency medicine departments of the IRCCS Policlinico Hospital, Milan, Italy, between April 2010 and January 2012. The institutional review board of the hospital approved the study (Approval reference number: 1,686) and patients signed an informed consent. Patients ≥18 years of age and satisfying the criteria of CAP were included in the study. Patients with at least one of the following were excluded from the study: hospital-acquired pneumonia (defined as pneumonia that develops after 48 h of the current hospitalisation), pneumonia that develops in patients discharged from the hospital within the prior 14 days, chronic steroid treatment, and unstable psychiatric or psychological conditions.

### Data collection

Patients’ management was performed according to local standard operating procedures. The following data were collected for each study subject on admission to hospital by one study investigator (LM): demographics (age, gender); comorbidities (active cancer, cerebrovascular disease, neurological disease, renal disease, liver disease, type II diabetes mellitus, chronic obstructive lung disease, hypertension, chronic heart failure, coronary artery disease, prior acute myocardial infarction); clinical signs and symptoms (body mass index, heart rate, respiratory rate, systolic blood pressure, diastolic blood pressure, oxygen saturation); radiographic and laboratory findings on admission and during hospitalisation (haematocrit, haemoglobin, white blood cells, platelets, albumin, lactates, C-reactive protein [CRP], procalcitonin [PCT]); time from symptoms onset to hospitalisation; empiric antibiotic therapy, according to the European Respiratory Society guidelines [14]; and severity of disease (pneumonia severity index [PSI] [15]), severe sepsis, severe CAP, alteration of gas exchange). The length of hospital stay (LOS) and in-hospital mortality were also noted for each patient. A paper case report form was used to collect anonymised data that have been then directly transferred to SPSS 18.0 for MAC OS (SPSS Inc., USA). Quality control of the data was performed by a study investigator (SA).

### Blood and EBC sample collection

Approximately 7 ml of blood and a specimen of EBC were collected within 24 h from hospital admission. After blood centrifugation, serum was stored at −80 °C. The EBC was collected for 15 min using the condensing device R-tube^TM^ (Cosmed, Italy). The collection was performed in compliance with the latest American Thoracic Society (ATS)/European Respiratory Society (ERS) recommendations [11]. Patients were asked to breathe out spontaneously through a mouthpiece and a two-way non-rebreathing valve, which also serves as a saliva trap, connected to the tube. Subjects wore a nose clip and were breathing with a respiratory rate that ranged from 15 to 20 breaths/min. If the subjects salivated they were instructed to swallow. Samples were stored at −80 °C for no longer than 4 weeks until measurements were taken.

### Microbiological analysis of respiratory samples

Microbiological examinations were performed on respiratory samples (e.g. sputum and tracheobronchial aspirates) during the first 24 h after admission and according to standard hospital protocols. Identification of microorganisms and susceptibility testing were performed according to standard methods [16].

### Cytokine measurements

Systemic and local inflammatory responses were evaluated measuring cytokines in both the serum and EBC samples with a high sensitivity immunoassay kit (Randox™, United Kingdom) and were classified as pro-inflammatory (interleukin [IL]-1, IL-1α, IL-1β, IL-2, IL-6, IL-8, tumor necrosis factor [TNF]α and interferon [IFN]γ) or anti-inflammatory (IL-4 and IL-10). The Evidence Investigator™ Cytokine & Growth Factors High Sensitivity Array (Randox) was used for the simultaneous quantitative detection of multiple cytokines from a single sample. The Randox Biochip (Randox) is a solid-state device containing an array of discrete test regions of immobilised antibodies specific to different cytokines and growth factors. A sandwich chemiluminescent immunoassay is employed for the cytokine array. Increased levels of cytokine in a specimen will lead to increased binding of antibody labeled with horseradish peroxidase, and thus an increase in the chemiluminescence signal emitted. The light signal generated from each of the test regions on the biochip is detected using digital imaging technology and compared to that from a stored calibration curve. The concentration of analytes present in the sample is calculated from the calibration curve.

### Study definitions

CAP was defined as the presence of a new pulmonary infiltrate on chest radiograph at the time of hospitalisation associated with at least one of the following: (i) new or increased cough with/without sputum production; (ii) fever (documented temperature, rectal or oral, ≥38.3 °C) or hypothermia (documented temperature, rectal or oral, <36 °C); (iii) evidence of systemic inflammation (abnormal white blood cell count—either leukocytosis [>10,000/cm^3^] or leukopenia [<4,000/cm^3^] or CRP or PCT values above the local upper limit).

Severe sepsis was defined by the presence of at least one of the following signs of organ hypoperfusion or organ dysfunction: (i) sepsis-induced hypotension; (ii) lactate greater than the upper limits of normal laboratory results; (iii) urine output <0.5 ml/kg/h for >2 h despite adequate fluid resuscitation; (iv) creatinine >2 mg/dl; (v) bilirubin >2 mg/dl; (vi) platelet count <100,000; (vii) coagulopathy (international normalised ratio [INR] >1.5) [17].

Severe CAP was defined according to the Infectious Diseases Society of America (IDSA)/ATS guidelines published in 2007 [18].

Alteration of gas exchange was defined as the presence of a PaO_2_/FiO_2_ ratio less than 250 with at least one of the following: (i) tachypnoea (respiratory rate >30 breaths/min); (ii) acute respiratory acidosis; or (iii) accessory muscle use.

LOS was calculated as the number of days from the date of admission to the date of discharge. LOS was censored at 14 days in an effort to capture only CAP-related LOS.

In-hospital mortality was considered if death by any cause occurred during hospitalisation.

### Study outcome

TCS was the study outcome. A patient was considered to have reached clinical stability when the following criteria were all met in a single day during hospitalisation: (i) improved clinical signs (cough and shortness of breath); (ii) patient was afebrile for at least 8 h; (iii) improving leukocytosis (decreased at least 10 % from the previous day) or CRP or PCT; (iv) tolerating oral intake [19]. Criteria for clinical stability were evaluated daily.

### Statistical analysis

Data were analysed using SPSS 18.0 for MAC OS (SPSS Inc., USA). Baseline characteristics of the population, biomarkers levels on admission, and outcome were considered for statistical analysis. Continuous variables are expressed as median (interquartile range [IQR] 25th–75th percentile). The difference of median (IQR) was evaluated by the Wilcoxon–Mann–Whitney U two-sample test. Categorical data are expressed as frequencies and percentages and compared using the Pearson’s chi-square or Fisher’s exact test where appropriate. Although similar comparisons have been performed multiple times in the analysis, we decided not to correct the level of significance to account for them, knowing that this would have reduced the chance of type I error. We opted for this choice in order to avoid increasing the chance of type II error. All tests were 2-tailed and a *p*-value of <0.05 was considered statistically significant.

## Results

### Study population characteristics

A total of 74 patients (median age: 76 years; gender: 61 % male) were enrolled during the study period. Demographics, comorbidities, physical examination, laboratory tests and severity of the disease on hospital admission of the study population are summarised in Tables 1 and 2. Aetiological diagnosis of CAP was reached in 10 (14 %) patients and the following microorganisms were the most commonly isolated: *Streptococcus pneumoniae* (6 patients), *Legionella pneumophila* (2 patients), and *Mycoplasma pneumoniae* (2 patients).

**Table 1:** Demographics and comorbidities of the study population (*n* = 74)

Characteristics	No. of patients
Demographics, *n* (%)	
Gender, male sex	45 (61)
Age, median years (IQR)	76 (65-83)
Comorbidities, *n* (%)	
Active cancer	8 (11)
Cerebrovascular diseases	7 (10)
Neurological diseases	16 (22)
Renal diseases	11 (15)
Liver diseases	6 (8)
Type II diabetes mellitus	14 (19)
COPD	21 (28)
Hypertension	36 (49)
Chronic heart failure	15 (20)
Coronary artery disease	19 (26)
Prior acute myocardial infarction	13 (18)

*IQR* 25–75 interquartile range, *COPD* chronic obstructive pulmonary disease

**Table 2:** Physical examination, laboratory tests and severity of the disease on hospital admission of patients with community-acquired pneumonia (CAP) (*n* = 74)

Characteristics	No. of patients
Physical examination, median (IQR)	
Body mass index, kg/m^2^	24.5 (21.0–27.5)
Heart rate, beats/minute	94 (80–112)
Respiratory rate, breaths/minute	24 (18–28)
Systolic blood pressure, mmHg	135 (113–160)
Diastolic Blood Pressure, mmHg	70 (60–85)
Oxygen saturation, %	95 (91–97)
Laboratory tests, median (IQR)	
Haematocrit, %	39 (34–42)
Haemoglobin, g/dl	12.8 (11.2–14.2)
White blood cells, 10^3^/ml	10.7 (8.2–14.2)
Platelets, 10^3^/ml	185 (149–258)
Albumin, g/dl	3.5 (3.2–3.8)
Lactates, mmol/l	1.2 (0.9–1.9)
C-reactive protein, mg/dl	11.4 (4.1–23.5)
Procalcitonin, ng/ml	1.0 (0.3–6.4)
Severity of the disease, *n* (%)	
PSI classes IV–V	58 (78)
Severe sepsis	20 (27)
Severe CAP	21 (28)
Alteration of gas exchange	10 (14)

*IQR* 25–75 interquartile range, *PSI* pneumonia severity index

Among the entire study population, median TCS was 4 days, while median LOS was 9 days. One patient died.

### Cytokine levels on hospital admission

Cytokine concentrations in both serum and EBC on hospital admission are shown in Table 3.

**Table 3:** Cytokine levels in both serum and exhaled breath condensate on hospital admission of patients with community-acquired pneumonia

Cytokine	Cytokine level (pg/ml)	
	Serum	Exhaled breath condensate
Pro-inflammatory		
IL-1	1.11 (0.87–1.53)	0.20 (0.08–1.10)
IL-1α	0.11 (0.06–0.21)	0.11 (0.05–0.22)
IL-1b	0.96 (0.76–1.36)	0.00 (0.00–0.95)
IL-2	1.14 (0.84–1.83)	0.80 (0.00–1.24)
IL-6	28.95 (9.92–106.27)	0.00 (0.00–0.10)
IL-8	12.82 (8.10–43.81)	0.36 (0.00–0.47)
IFNγ	0.82 (0.46–2.81)	0.00 (0.00–0.16)
TNFα	5.86 (3.85–8.94)	0.00 (0.00–0.76)
Anti-inflammatory		
IL-4	1.53 (1.32–1.99)	1.98 (1.55–2.39)
IL-10	1.33 (0.78–2.08)	0.27 (0.00–0.37)

Data presented as median (interquartile range)

*IL* interleukin, *IFN* interferon, *TNF* tumor necrosis factor

### Cytokine levels and severity of the disease on hospital admission

A complete analysis of cytokine levels according to the severity of the disease on hospital admission is presented in the Additional file 1: Table SA). Briefly, levels of IL-1α were found to be significantly higher in both serum and EBC of patients in PSI Risk Class IV–V versus I–III. Levels of IL-1 and IL-1α were found to be significantly higher both in serum and EBC of patients with altered gas exchange compared to those without. TNF-α levels in serum and IL-1β levels in the EBC were significantly higher in patients with altered gas exchange compared to those without. IL-6 and IL-8 levels were found to be significantly higher in the serum of patients with severe sepsis compared to those without, while IL-10 was significantly lower in the EBC of patients with both severe sepsis and severe CAP, compared to those without.

As shown in Table 4 (Panel A and Panel B), anti- to pro-inflammatory cytokine ratios were found to be significantly lower in patients with severe CAP and severe sepsis. The lower ratio in serum of patients with severe infection was mainly due to a more active pro-inflammatory profile (significantly higher levels of IL-6 and IL-8; *p* < 0.006 and *p* <0.004, respectively [Additional file 1: Table SA]). On the contrary, the lower ratio in the EBC of patients with severe infection was mainly due to a less intense anti-inflammatory profile (significantly lower levels of IL-10; *p* < 0.017 [Additional file 1: Table SA]). A complete analysis of cytokine ratios according to the severity of disease on admission is presented in Additional file 1: Table SB.

**Table 4:** Significant comparisons between anti- to pro-inflammatory cytokine ratios according to the severity of the disease (Panel A) and the presence of severe sepsis (Panel B) in patients with community-acquired pneumonia (CAP)

Cytokine ratio	Cytokine level (pg/ml)		*p*-value
Panel A			
	Severe CAP	Non severe CAP	
Serum			
IL-4/IL-8	0.03 (0.01–0.15)	0.12 (0.04–0.22)	0.038
IL-4/IFNγ	0.90 (0.02–2.16)	1.64 (0.31–4.09)	0.047
IL-4/IL-1	1.20 (0.32–1.35)	1.60 (1.15–2.34)	0.001
IL-4/IL-1b	1.26 (0.47–1.77)	1.60 (1.20–2.24)	0.035
IL-10/IL-8	0.05 (0.02–0.16)	0.12 (0.05–0.20)	0.048
Exhaled breath condensate			
IL-4/IL-1b	1.94 (1.72–2.26)	2.37 (2.02–3.36)	0.044
IL-10/IL-2	0.00 (0.00–0.29)	0.35 (0.25–0.47)	0.005
IL-10/IL-6	0.00 (0.00–0.00)	3.02 (0.58–3.82)	0.016
IL-10/IL-8	0.00 (0.00–0.41)	0.75 (0.19–1.72)	0.002
Panel B			
	Severe sepsis	Non severe sepsis	
Serum			
IL-4/IL-6	0.01 (0.00–0.04)	0.07 (0.02–0.17)	0.000
IL-4/IL-8	0.03 (0.00–0.10)	0.12 (0.06–0.21)	0.002
IL-4/IL-1	1.23 (0.15–1.61)	1.55 (1.10–2.32)	0.006
IL-10/IL-6	0.01 (0.00–0.07)	0.05 (0.02–0.12)	0.017
IL-10/IL-8	0.03 (0.01–0.18)	0.12 (0.05–0.19)	0.039
Exhaled breath condensate			
IL-10/IL-2	0.00 (0.00–0.21)	0.38 (0.26–0.48)	0.000

Data presented as median (interquartile range)

*IL* interleukin, *IFN* interferon

### Cytokine levels and clinical outcome

IL-6 levels were higher among patients with TCS >4 days versus TCS ≤4 days, especially in serum (43 vs. 25 pg/ml; *p* = 0.010) (see text and Additional file 1: Table SC). Anti- to pro-inflammatory cytokine ratios were found to be significantly lower in patients with a TCS >4 days. Median (IQR) IL-4/IL-6 ratio in serum was 0.02 (0.01–0.10) pg/ml in patients with a TCS >4 days versus 0.07 (0.02–0.20) pg/ml in those with a TCS ≤4 days (*p* = 0.027). Median (IQR) IL-4/IL-8 ratio in serum was 0.04 (0.03–0.13) pg/ml in those with a TCS > 4 days versus 0.12 (0.06–0.27) pg/ml in those with a TCS ≤4 days; *p* = 0.029. A complete analysis of cytokine levels and ratios according to TCS is presented in Additional file 1: Table SC.

## Discussion

The present study indicates differences in inflammatory patterns in the EBC and serum on hospital admission in patients with CAP, with a high anti-inflammatory IL-4 concentration in the EBC and a high pro-inflammatory IL-6 and IL-8 concentration in serum. The anti- to pro-inflammatory cytokine ratio is decreased in patients with both severe disease on admission and prolonged TCS. This reduction seems to be due to lower levels of anti-inflammatory cytokines in the EBC and higher levels of pro-inflammatory cytokines in serum.

Local concentrations of both anti- and pro-inflammatory cytokines were evaluated in the present study using the EBC. The EBC is a standardised, non-invasive technique that has been extensively used to collect airway lining fluid and assess lung inflammation in several respiratory diseases during the past two decades [11]. To date, only one study compared systemic to local inflammatory response by measuring cytokines in peripheral blood and EBC in patients with pneumonia [20]. In this latest study, authors compared levels of TNFα and vascular endothelial growth factor in patients with pneumonia and lung cancer, as well as healthy controls, while we evaluated a wide range of cytokines in a homogenous model such as CAP. One of the major limitations of this technique is that in the absence of a comparison with BAL results we might not be fully confident that EBC results are in fact representative of the real inflammation of the parenchyma, rather than inflammation of the airways.

Our analysis showed some differences in patterns of cytokine expression in serum and EBC. However, the fact that these are two different biological samples, along with the absence of a control group in our study, dose not allow us to make strong speculation on the possible presence of two different compartments of the host inflammatory response (lung and blood). We found high concentrations of the anti-inflammatory IL-4 in EBC on admission, while in serum pro-inflammatory activation was prominent. An anti-inflammatory activation in the lungs, through the expression of the anti-inflammatory IL-10, has already been reported by van der Poll and colleagues [21] in a mouse model of *S. pneumoniae* pneumonia.

High levels of serum pro-inflammatory cytokines showed a direct relation with the severity of the disease on admission. These data support the hypothesis that severe infection is a consequence of the dis-regulation between pro- and anti-inflammatory pathways [8, 22]. Our results are also consistent with previous observations [9, 23], which describe an association between systemic levels of pro-inflammatory cytokines and severity of CAP. Paats et al. [23] found that in the BAL of patients with severe CAP, both IL-6 and IFN-γ were significantly increased compared with healthy individuals. Similarly, we found higher levels of the pro-inflammatory cytokine IL-1β in the EBC of patients with altered gas exchange and lower levels of the anti-inflammatory cytokine IL-10 in the EBC of patients with severe sepsis and severe CAP.

Despite a consistent number of observations, the role of pro- and anti-inflammatory mediators considered on their own is still unclear. Pro-inflammatory molecules are part of the host defence mechanisms against infection, but they also play a role in the “cytokine storm” that may trigger severe sepsis and acute respiratory distress syndrome. Similarly, anti-inflammatory molecules, at an early stage, may hamper the effective clearance of the infection, but they also have a role in the modulation and prevention of an excessive immune response. In this scenario, what seems to be important is the relationship between anti- and pro-inflammatory mediators, which can be evaluated through the anti-/pro-inflammatory cytokine ratios. An adequate response to infection should aim at a timely balance between the two, while an imbalance leads to an inadequate or excessive host defensive response. Our results are consistent with this hypothesis. The anti-/pro-inflammatory cytokine ratios were significantly lower both in serum and EBC of patients with severe CAP and severe sepsis, compared to patients without severe infection. Alterations in cytokines ratio were also found in patients who showed a prolonged TCS compared to patients with a shorter one. Similar results were reported by Chou and colleagues [13] in 25 patients with *P. jirovecii* pneumonia: a pro-inflammatory imbalance in the pro-/anti-inflammatory cytokine ratio was described in the BAL of patients with more severe respiratory failure and worse clinical outcomes. It is noteworthy that in our patients with severe infection the lower serum ratio was mainly due to a more active pro-inflammatory profile, while the lower EBC ratio was mainly due to a less intense anti-inflammatory reaction.

Different limitations of the present study should be acknowledged, including: (i) the absence of a control group without CAP to better interpret cytokine values in the EBC; (ii) the fact that measurements from EBC were not compared with results from BAL and, thus, EBC results might not be fully representative of the extent of inflammation in the lungs; (iii) the absence of timing of sample collection (EBC and serum were collected only on hospital admission) and therefore not allowing a longitudinal evaluation of the inflammatory patterns; (iv) the isolation of the microorganism causing pneumonia only in a small percentage of patients, not allowing any speculation regarding the association between pathogen and inflammatory response; (v) missing data, in view of the small sample size, regarding the relationship between cytokines profile and other early and long-term outcomes, such as mortality during hospitalisation.

Our study is strengthened by the use of EBC instead of BAL, which allowed us to enrol patients from mild to severe CAP. This aspect and the fact that we did not use important exclusion criteria increase the generalisability of our results. Furthermore, we conducted a comprehensive evaluation of cytokines, including both anti- and pro-inflammatory mediators.

## Conclusions

In conclusion, CAP patients with severe disease on hospital admission and prolonged TCS showed an imbalance in the pro-/anti-inflammatory cytokines ratio toward a pro-inflammatory profile. A deeper knowledge of the pro- and anti-inflammatory mechanisms may help to evaluate new therapeutic approaches.

## Abbreviations

ATS, American Thoracic Society; BAL, bronchoalveolar lavage; CAP, community-acquired pneumonia; CRP, C-reactive protein; EBC, exhaled breath condensate; ERS, European Respiratory Society; IDSA, Infectious Diseases Society of America; IFN, interferon; IL, interleukin; INR, international normalized ratio; IQR, interquartile range; LOS, length of hospital stay; PCT, procalcitonin; PSI, pneumonia severity index; TCS, time to clinical stability; TNF, tumor necrosis factor

## Additional file

Additional file 1: Table SA. Complete analysis of cytokine levels in serum and exhaled breath condensate according to the severity of the disease on hospital admission of patients with community-acquired pneumonia (CAP). Table SB. Complete analysis of cytokine ratio according to the severity of the disease on hospital admission of patients with community-acquired pneumonia (CAP). Table SC. Complete analysis of cytokine levels and ratios in serum and exhaled breath condensate according to time to clinical stability (TCS). (DOC 275 kb)
