# Supplementary material for: Serum and exhaled breath condensate inflammatory cytokines in community-acquired pneumonia: a prospective cohort study
Source: Pneumonia (Nathan). 2016 Jun 23;8:8. doi: 10.1186/s41479-016-0009-7 (PMC5471930; doi:10.1186/s41479-016-0009-7)
Supplement: Additional file 1: Table SA. — Complete analysis of cytokine levels in serum and exhaled breath condensate according to the severity of the disease on hospital admission of patients with community-acquired pneumonia (CAP). Table SB. Complete analysis of cytokine ratio according to the severity of the disease on hospital admission of patients with community-acquired pneumonia (CAP). Table SC. Complete analysis of cytokine levels and ratios in serum and exhaled breath condensate according to time to clinical stability (TCS). (DOC 275 kb) [file 41479_2016_9_MOESM1_ESM.doc]

**Additional file**

**RESULTS**

**Cytokine measurements**

**Cytokine levels and severity of the disease on hospital admission**

Median (25-75 interquartile range [IQR]) levels of IL-1α were found to be significantly higher in serum of patients in pneumonia severity index (PSI) Risk Class IV–V *versus* I–III: 0.12 (0.07–0.22) pg/ml *versus* 0.085 (0.05–0.13) pg/ml; *p* = 0.042. Similar results of IL-1α for PSI Risk Class IV–V *versus* I–III were found in the exhaled breath condensate (EBC): 0.11 (0.06–0.22) pg/ml *versus* 0.06 (0.04–0.11) pg/ml; *p* = 0.028.

Median (IQR) levels of IL-1 and IL-1α were found to be significantly higher both in serum and EBC of patients with altered gas exchange compared to those without. IL-1 levels: 1.52 (1.06–3.74) *versus* 1.09 (0.81–1.48) pg/ml; *p* = 0.041, in serum and 1.07 (0.84–1.19) *versus* 0.13 (0.05–1.05) pg/ml; *p* = 0.009, in the EBC. IL-1α levels: 0.19 (0.10–0.57) *versus* 0.11 (0.05–0.20) pg/ml; *p* = 0.025, in serum and 0.19 (0.15–0.27) *versus* 0.10 (0.05–0.16) pg/ml; *p* = 0.003, in the EBC.

TNF-α levels in serum were higher in patients with altered gas exchange (8.74 pg/ml) compared to those without (5.62 pg/ml); *p* = 0.040; and IL-1β levels in the EBC were also higher in patients with altered gas exchange (0.74 pg/ml) compared to those without (0 pg/ml).

Median (IQR) levels of two pro-inflammatory cytokines were found to be significantly higher in the serum of patients with severe sepsis compared to those without: IL-6 (64.89 [26.43–386.17] pg/ml *versus* 27.19 (9.13–77.21) pg/ml; *p* = 0.006) and IL-8 (47.30 [10.53-89.84] pg/ml *versus* 11.45 [7.57-26.82] pg/ml; *p* = 0.004). On the other hand, the median (IQR) levels of the anti-inflammatory cytokine IL-10 was significantly lower in the EBC of patients with both severe sepsis (0.09 [0.00–0.31] pg/ml vs. 0.34 [0.00–0.40] pg/ml; *p* = 0.017) and severe CAP (0.00 [0.00–0.27] pg/ml vs. 0.35 [0.00–0.40] pg/ml; *p* = 0.001) compared to those without. A complete analysis of cytokine levels according to the severity on admission is presented in Table A

**Cytokine levels and time to clinical stability (TCS)**

A complete analysis of cytokine levels and ratios according to TCS is presented in Table C. Median (IQR) levels of IL-6 were higher among patients with a longer TCS in both serum and EBC: serum = 43.16 (20.06–171.31) pg/ml in patients with TCS >4 days *versus* 25.86 (8.60–57.26) pg/ml in TCS ≤4 days; *p* = 0.010; EBC = 0.00 (0.00–0.11) pg/ml in patients with TCS >4 days *versus* 0.00 (0.00–0.05) pg/ml in TCS ≤ 4 days; *p* = 0.039. Anti- to pro-inflammatory cytokine ratios were found to be significantly lower in patients with a TCS >4 days. Median (IQR) IL-4/IL-6 ratio in serum was 0.02 (0.01–0.10) pg/ml in patients with a TCS >4 days *versus* 0.07 (0.02–0.20) pg/ml in those with a TCS ≤4 days; *p* = 0.027. Median (IQR) IL-4/IL-8 ratio in serum was 0.04 (0.03–0.13) pg/ml in those with a TCS >4 days *versus* 0.12 (0.06–0.27) pg/ml in those with a TCS ≤4 days; *p* = 0.029.

**TABLES**

Table SA. Complete analysis of cytokine levels in serum and exhaled breath condensate according to the severity of the disease on hospital admission of patients with community-acquired pneumonia (CAP)

| **Cytokine** | **Cytokine level (pg/ml)** | | ***p*-value** |
| --- | --- | --- | --- |
|  | **PSI I-III** | **PSI IV-V** |  |
| **Serum** | | | |
| IL-2 | 1.10 (0.90-1.42) | 1.17 (0.82-1.86) | 0.489 |
| IL-4 | 1.47 (1.23-1.71) | 1.54 (1.34-2.15) | 0.264 |
| IL-6 | 17.86 (4.33-76.33) | 31.05 (13.40-123.36) | 0.078 |
| IL-8 | 9.11 (6.28-12.59) | 17.81 (8.40-45.59) | 0.060 |
| IL-10 | 1.03 (0.56-1.57) | 1.40 (0.85-2.22) | 0.114 |
| IFNγ | 0.76 (0.51-7.07) | 0.84 (0.43-2.57) | 0.783 |
| TNFα | 4.67 (1.69-6.50) | 6.02 (4.14-9.57) | 0.060 |
| IL-1 | 1.03 (0.86-1.30) | 1.14 (0.85-1.60) | 0.435 |
| IL-1a | 0.09 (0.05-0.13) | 0.12 (0.07-0.22) | 0.042 |
| IL-1b | 0.96 (0.79-1.23) | 0.96 (0.73-1.38) | 0.854 |
| **Exhaled breath condensate** | | | |
| IL-2 | 0.81 (0.00-1.07) | 0.79 (0.00-1.34) | 0.746 |
| IL-4 | 1.97 (1.63-2.19) | 2.03 (1.47-2.61) | 0.382 |
| IL-6 | 0.00 (0.00-0.07) | 0.00 (0.00-0.10) | 0.148 |
| IL-8 | 0.43 (0.00-0.48) | 0.35 (0.00-0.46) | 0.828 |
| IL-10 | 0.24 (0.00-0.37) | 0.31 (0.00-0.38) | 0.537 |
| IFNγ | 0.00 (0.00-0.23) | 0.00 (0.00-0.16) | 0.673 |
| TNFα | 0.00 (0.00-0.76) | 0.00 (0.00-0.76) | 0.956 |
| IL-1 | 0.11 (0.05-1.00) | 0.27 (0.09-1.11) | 0.370 |
| IL-1a | 0.06 (0.04-0.11) | 0.11 (0.06-0.22) | 0.028 |
| IL-1b | 0.00 (0.00-0.8) | 0.00 (0.00-0.95) | 0.577 |
|  |  |  |  |
|  | **Normal gas exchange** | **Altered gas exchange** |  |
| **Serum** | | | |
| IL-2 | 1.13 (0.82-1.75) | 1.57 (1.03-2.75) | 0.219 |
| IL-4 | 1.54 (1.31-1.88) | 1.52 (1.29-2.41) | 0.602 |
| IL-6 | 28.26 (9.86-100.12) | 44.32 (12.76-245.93) | 0.212 |
| IL-8 | 12.08 (8.08-34.60) | 36.94 (9.02-245.70) | 0.155 |
| IL-10 | 1.27 (0.67-2.04) | 1.58 (1.21-2.93) | 0.133 |
| IFNγ | 0.82 (0.48-2.84) | 0.75 (0.36-2.45) | 0.402 |
| TNFα | 5.62 (3.19-8.56) | 8.74 (5.55-24.03) | 0.040 |
| IL-1 | 1.09 (0.81-1.48) | 1.52 (1.06-3.74) | 0.041 |
| IL-1a | 0.11 (0.05-0.20) | 0.19 (0.10-0.57) | 0.025 |
| IL-1b | 0.91 (0.72-1.29) | 1.04 (0.95-2.81) | 0.096 |
| **Exhaled breath condensate** | | | |
| IL-2 | 0.79 (0.00-1.17) | 1.01 (0.59-1.75) | 0.076 |
| IL-4 | 2.02 (1.57-2.40) | 1.69 (1.46-2.62) | 0.513 |
| IL-6 | 0.00 (0.00-0.10) | 0.05 (0.00-0.15) | 0.357 |
| IL-8 | 0.33 (0.00-0.46) | 0.44 (0.28-0.67) | 0.093 |
| IL-10 | 0.25 (0.00-0.37) | 0.31 (0.00-0.52) | 0.418 |
| IFNγ | 0.00 (0.00-0.21) | 0.00 (0.00-0.00) | 0.174 |
| TNFα | 0.00 (0.00-0.71) | 0.00 (0.00-0.77) | 0.906 |
| IL-1 | 0.13 (0.05-1.05) | 1.07 (0.84-1.19) | 0.009 |
| IL-1a | 0.10 (0.05-0.16) | 0.19 (0.15-0.27) | 0.003 |
| IL-1b | 0.00 (0.00-0.92) | 0.74 (0.63-0.96) | 0.040 |
|  |  |  |  |
|  | **Non severe sepsis** | **Severe sepsis** |  |
| **Serum** | | | |
| IL-2 | 1.15 (0.82-1.86) | 1.13 (0.88-1.74) | 0.971 |
| IL-4 | 1.54 (1.34-2.00) | 1.52 (0.99-1.99) | 0.511 |
| IL-6 | 27.19 (9.13-77.21) | 64.89 (26.43-386.17) | 0.006 |
| IL-8 | 11.45 (7.57-26.82) | 47.30 (10.53-89.84) | 0.004 |
| IL-10 | 1.24 (0.72-2.04) | 1.61 (0.95-2.13) | 0.312 |
| IFNγ | 0.85 (0.47-2.81) | 0.68 (0.42-4.97) | 0.817 |
| TNFα | 5.57 (3.55-8.41) | 5.94 (5.00-11.94) | 0.268 |
| IL-1 | 1.09 (0.84-1.48) | 1.17 (0.96-1.85) | 0.271 |
| IL-1a | 0.12 (0.07-0.22) | 0.09 (0.05-0.20) | 0.613 |
| IL-1b | 0.91 (0.73-1.27) | 1.04 (0.85-1.77) | 0.177 |
| **Exhaled breath condensate** | | | |
| IL-2 | 0.79 (0.00-1.19) | 0.82 (0.00-1.31) | 0.659 |
| IL-4 | 1.98 (1.46-2.39) | 2.02 (1.61-2.57) | 0.795 |
| IL-6 | 0.00 (0.00-0.09) | 0.00 (0.00-0.10) | 0.688 |
| IL-8 | 0.37 (0.00-0.49) | 0.18 (0.00-0.44) | 0.380 |
| IL-10 | 0.34 (0.00-0.40) | 0.09 (0.00-0.31) | 0.017 |
| IFNγ | 0.00 (0.00-0.23) | 0.00 (0.00-0.00) | 0.312 |
| TNFα | 0.00 (0.00-0.76) | 0.00 (0.00-0.00) | 0.319 |
| IL-1 | 0.17 (0.10-1.07) | 0.46 (0.04-1.12) | 0.630 |
| IL-1a | 0.11 (0.06-0.22) | 0.10 (0.04-0.21) | 0.334 |
| IL-1b | 0.00 (0.00-0.86) | 0.32 (0.00-0.99) | 0.735 |
|  |  |  |  |
|  | **Non severe CAP** | **Severe CAP** |  |
| **Serum** | | | |
| IL-2 | 1.17 (0.86-1.79) | 1.07 (0.81-2.06) | 0.909 |
| IL-4 | 1.54 (1.34-2.05) | 1.48 (1.12-1.88) | 0.325 |
| IL-6 | 28.02 (9.85-96.82) | 36.02 (13.88-346.11) | 0.170 |
| IL-8 | 11.90 (7.46-28.99) | 29.32 (8.54-79.56) | 0.067 |
| IL-10 | 1.30 (0.90-2.07) | 1.58 (0.55-2.09) | 0.843 |
| IFNγ | 0.75 (0.43-2.44) | 1.23 (0.55-6.12) | 0.278 |
| TNFα | 6.00 (3.81-8.75) | 5.84 (3.42-9.61) | 0.971 |
| IL-1 | 1.09 (0.86-1.52) | 1.17 (0.87-1.64) | 0.308 |
| IL-1a | 0.11 (0.07-0.21) | 0.12 (0.06-0.21) | 0.723 |
| IL-1b | 0.91 (0.76-1.32) | 1.04 (0.78-1.56) | 0.251 |
| **Exhaled breath condensate** | | | |
| IL-2 | 0.79 (0.00-1.27) | 0.82 (0.00-1.22) | 0.890 |
| IL-4 | 1.98 (1.49-2.39) | 1.92 (1.58-2.49) | 0.939 |
| IL-6 | 0.00 (0.00-0.10) | 0.00 (0.00-0.10) | 0.731 |
| IL-8 | 0.37 (0.00-0.46) | 0.00 (0.00-0.49) | 0.634 |
| IL-10 | 0.35 (0.00-0.40) | 0.00 (0.00-0.27) | 0.001 |
| IFNγ | 0.00 (0.00-0.23) | 0.00 (0.00-0.08) | 0.584 |
| TNFα | 0.00 (0.00-0.66) | 0.00 (0.00-0.76) | 0.980 |
| IL-1 | 0.27 (0.10-1.06) | 0.15 (0.05-1.16) | 0.617 |
| IL-1a | 0.11 (0.06-0.22) | 0.1 (0.05-0.19) | 0.348 |
| IL-1b | 0.00 (0.00-0.89) | 0.00 (0.00-0.98) | 0.934 |

Data presented as median (interquartile range)

PSI, pneumonia severity index; IL, interleukin; IFN, interferon; TNF, tumour necrosis factor

Table SB. Complete analysis of cytokine ratio according to the severity of the disease on hospital admission of patients with community-acquired pneumonia (CAP)

| **Cytokine ratio** | **Cytokine level (pg/ml)** | | ***p*-value** |
| --- | --- | --- | --- |
|  | **Non severe sepsis** | **Severe sepsis** |  |
| **Serum** | | | |
| IL4 / IL2 | 1.23 (0.93-1.43) | 1.38 (0.34-1.64) | 0.980 |
| IL4 / IL6 | 0.07 (0.02-0.17) | 0.01 (0.00-0.04) | 0.000 |
| IL4 / IL8 | 0.12 (0.06-0.21) | 0.03 (0.00-0.10) | 0.002 |
| IL4 / IFNγ | 1.41 (0.23-3.71) | 1.78 (0.01-3.38) | 0.524 |
| IL4 / TNFα | 0.30 (0.15-0.45) | 0.15 (0.03-0.34) | 0.010 |
| IL4 / IL1 | 1.55 (1.10-2.32) | 1.23 (0.15-1.61) | 0.006 |
| IL4 / IL1a | 11.32 (8.59-17.1) | 10.89 (0.30-22.62) | 0.649 |
| IL4 / IL1b | 1.59 (1.15-2.23) | 1.34 (0.23-1.86) | 0.057 |
| IL10 / IL2 | 0.84 (0.48-1.66) | 1.23 (0.67-1.87) | 0.341 |
| IL10 / IL6 | 0.05 (0.02-0.12) | 0.01 (0.00-0.07) | 0.017 |
| IL10 / IL8 | 0.12 (0.05-0.19) | 0.03 (0.01-0.18) | 0.039 |
| IL10 / IFNγ | 1.02 (0.36-2.66) | 2.00 (0.21-3.41) | 0.463 |
| IL10 / TNFα | 0.25 (0.13-0.34) | 0.18 (0.09-0.44) | 0.721 |
| IL10 / IL1 | 1.11 (0.73-3.59) | 1.46 (0.90-1.96) | 0.959 |
| IL10 / IL1a | 12.25 (4.61-20.14) | 13.29 (5.91-27.26) | 0.514 |
| IL10 / IL1b | 1.08 (0.58-1.84) | 1.48 (0.83-2.12) | 0.293 |
| **Exhaled breath condensate** | | | |
| IL4 / IL2 | 1.84 (1.59-2.11) | 1.79 (1.28-2.70) | 0.718 |
| IL4 / IL6 | 17.56 (15.67-23.15) | 15.51 (13.41-20.23) | 0.198 |
| IL4 / IL8 | 4.30 (2.85-6.52) | 4.24 (3.45-8.92) | 0.623 |
| IL4 / IFNγ | 8.61 (7.72-9.78) | 7.19 (6.30-9.15) | 0.178 |
| IL4 / TNFα | 2.36 (1.86-2.76) | 2.17 (2.10-2.64) | 0.961 |
| IL4 / IL1 | 6.91 (1.83-20.60) | 2.52 (1.57-43.18) | 0.609 |
| IL4 / IL1a | 17.84 (10.41-24.92) | 16.33 (10.96-43.18) | 0.800 |
| IL4 / IL1b | 2.29 (1.95-3.24) | 2.03 (1.66-3.01) | 0.343 |
| IL10 / IL2 | 0.38 (0.26-0.48) | 0.00 (0.00-0.21) | 0.000 |
| IL10 / IL6 | 3.04 (0.00-3.89) | 0.00 (0.00-2.40) | 0.045 |
| IL10 / IL8 | 0.69 (0.03-1.22) | 0.00 (0.00-0.78) | 0.098 |
| IL10 / IFNγ | 1.48 (0.31-2.31) | 0.72 (0.00-1.72) | 0.385 |
| IL10 / TNFα | 0.40 (0.00-0.47) | 0.00 (0.00-0.32) | 0.262 |
| IL10 / IL1 | 0.50 (0.00-2.99) | 0.00 (0.00-0.47) | 0.026 |
| IL10 / IL1a | 2.50 (0.00-3.66) | 0.00 (0.00-2.29) | 0.017 |
| IL10 / IL1b | 0.45 (0.00-0.68) | 0.17 (0.00-0.50) | 0.207 |
|  |  |  |  |
|  | **Non severe CAP** | **Severe CAP** |  |
| **Serum** | | | |
| IL4 / IL2 | 1.28 (0.93-1.45) | 1.17 (0.39-1.46) | 0.333 |
| IL4 / IL6 | 0.05 (0.02-0.14) | 0.02 (0.00-0.08) | 0.051 |
| IL4 / IL8 | 0.12 (0.04-0.22) | 0.03 (0.01-0.15) | 0.038 |
| IL4 / IFNγ | 1.64 (0.31-4.09) | 0.90 (0.02-2.16) | 0.047 |
| IL4 / TNFα | 0.29 (0.14-0.46) | 0.23 (0.12-0.37) | 0.280 |
| IL4 / IL1 | 1.60 (1.15-2.34) | 1.20 (0.32-1.35) | 0.001 |
| IL4 / IL1a | 11.66 (8.96-19.21) | 8.59 (5.33-13.11) | 0.111 |
| IL4 / IL1b | 1.60 (1.20-2.24) | 1.26 (0.47-1.77) | 0.035 |
| IL10 / IL2 | 0.94 (0.55-1.66) | 1.13 (0.40-1.92) | 0.935 |
| IL10 / IL6 | 0.04 (0.02-0.13) | 0.03 (0.01-0.07) | 0.086 |
| IL10 / IL8 | 0.12 (0.05-0.20) | 0.05 (0.02-0.16) | 0.048 |
| IL10 / IFNγ | 1.59 (0.59-3.24) | 0.82 (0.20-2.00) | 0.055 |
| IL10 / TNFα | 0.25 (0.11-0.38) | 0.19 (0.11-0.36) | 0.692 |
| IL10 / IL1 | 1.15 (0.78-3.90) | 1.24 (0.40-1.95) | 0.271 |
| IL10 / IL1a | 13.54 (5.10-21.25) | 10.40 (4.25-17.00) | 0.383 |
| IL10 / IL1b | 1.16 (0.76-1.90) | 1.24 (0.49-2.15) | 0.882 |
| **Exhaled breath condensate** | | | |
| IL4 / IL2 | 1.84 (1.49-2.13) | 1.81 (1.60-2.56) | 0.686 |
| IL4 / IL6 | 17.13 (15.41-22.86) | 16.82 (13.92-21.10) | 0.607 |
| IL4 / IL8 | 4.20 (2.85-7.91) | 4.60 (3.54-6.36) | 0.669 |
| IL4 / IFNγ | 8.61 (7.13-9.13) | 8.59 (6.69-9.88) | 0.866 |
| IL4 / TNFα | 2.48 (1.91-2.82) | 2.16 (1.98-2.43) | 0.467 |
| IL4 / IL1 | 4.03 (1.97-19.80) | 3.46 (1.53-28.40) | 0.887 |
| IL4 / IL1a | 17.63 (10.52-24.19) | 17.60 (10.67-28.40) | 0.523 |
| IL4 / IL1b | 2.37 (2.02-3.36) | 1.94 (1.72-2.26) | 0.044 |
| IL10 / IL2 | 0.35 (0.25-0.47) | 0.00 (0.00-0.29) | 0.005 |
| IL10 / IL6 | 3.02 (0.58-3.82) | 0.00 (0.00-0.00) | 0.016 |
| IL10 / IL8 | 0.75 (0.19-1.72) | 0.00 (0.00-0.41) | 0.002 |
| IL10 / IFNγ | 1.46 (0.00-2.31) | 1.22 (0.00-2.19) | 0.672 |
| IL10 / TNFα | 0.41 (0.00-0.45) | 0.00 (0.00-0.41) | 0.244 |
| IL10 / IL1 | 0.50 (0.00-2.96) | 0.00 (0.00-1.14) | 0.039 |
| IL10 / IL1a | 2.96 (0.81-3.66) | 0.00 (0.00-2.07) | 0.007 |
| IL10 / IL1b | 0.51 (0.29-0.73) | 0.00 (0.00-0.35) | 0.005 |

Data presented as median (interquartile range)

IL, interleukin; IFN, interferon; TNF, tumour necrosis factor

Table SC. Complete analysis of cytokine levels and ratios in serum and exhaled breath condensate according to time to clinical stability (TCS)

| **Cytokine** | **Cytokine level (pg/ml)** | | ***p*-value** |
| --- | --- | --- | --- |
|  | **TCS ≤4 days** | **TCS >4 days** |  |
| **Serum** | | | |
| IL-2 | 1.27 (0.94-1.83) | 1.12 (0.82-1.83) | 0.394 |
| IL-4 | 1.54 (1.32-2.22) | 1.48 (1.31-1.86) | 0.436 |
| IL-6 | 25.86 (8.60-57.26) | 43.16 (20.06-171.31) | 0.010 |
| IL-8 | 10.04 (7.11-19.77) | 33.51 (10.23-68.91) | 0.004 |
| IL-10 | 1.28 (0.64-2.09) | 1.41 (0.89-2.04) | 0.360 |
| IFNγ | 0.64 (0.41-2.79) | 1.23 (0.60-6.09) | 0.089 |
| TNFα | 5.80 (3.73-8.84) | 6.00 (4.00-9.18) | 0.784 |
| IL-1 | 1.11 (0.88-1.55) | 1.10 (0.66-1.48) | 0.599 |
| IL-1a | 0.10 (0.05-0.20) | 0.13 (0.07-0.22) | 0.392 |
| IL-1b | 0.97 (0.83-1.37) | 0.87 (0.60-1.35) | 0.376 |
| **Exhaled breath condensate** | | | |
| IL-2 | 0.82 (0.00-1.38) | 0.79 (0.00-1.12) | 0.317 |
| IL-4 | 1.92 (1.60-2.51) | 2.08 (1.44-2.32) | 0.938 |
| IL-6 | 0.00 (0.00-0.11) | 0.00 (0.00-0.05) | 0.039 |
| IL-8 | 0.37 (0.00-0.46) | 0.00 (0.00-0.49) | 0.520 |
| IL-10 | 0.31 (0.00-0.41) | 0.23 (0.00-0.37) | 0.318 |
| IFNγ | 0.00 (0.00-0.20) | 0.00 (0.00-0.16) | 0.648 |
| TNFα | 0.00 (0.00-0.76) | 0.00 (0.00-0.00) | 0.373 |
| IL-1 | 0.67 (0.09-1.12) | 0.11 (0.06-0.88) | 0.310 |
| IL-1a | 0.11 (0.05-0.22) | 0.10 (0.05-0.19) | 0.590 |
| IL-1b | 0.63 (0.00-0.98) | 0.00 (0.00-0.75) | 0.275 |
|  |  |  |  |
| **Cytokine ratio** |  |  |  |
| **Serum** | | | |
| IL4 / IL2 | 1.18 (0.92-1.43) | 1.28 (0.79-1.53) | 0.770 |
| IL4 / IL6 | 0.07 (0.02-0.20) | 0.02 (0.01-0.10) | 0.027 |
| IL4 / IL8 | 0.12 (0.06-0.27) | 0.04 (0.03-0.13) | 0.029 |
| IL4 / IFNγ | 1.68 (0.15-3.82) | 1.29 (0.23-3.38) | 0.610 |
| IL4 / TNFα | 0.28 (0.13-0.39) | 0.23 (0.15-0.39) | 0.911 |
| IL4 / IL1 | 1.44 (1.07-1.83) | 1.49 (0.96-2.21) | 0.689 |
| IL4 / IL1a | 11.78 (7.74-17.55) | 10.60 (6.18-18.73) | 0.686 |
| IL4 / IL1b | 1.52 (1.23-2.01) | 1.55 (0.84-1.98) | 0.443 |
| IL10 / IL2 | 0.89 (0.48-1.48) | 1.13 (0.61-1.97) | 0.258 |
| IL10 / IL6 | 0.05 (0.02-0.14) | 0.03 (0.01-0.06) | 0.064 |
| IL10 / IL8 | 0.11 (0.05-0.20) | 0.05 (0.02-0.15) | 0.154 |
| IL10 / IFNγ | 1.12 (0.41-3.17) | 1.34 (0.21-2.60) | 0.676 |
| IL10 / TNFα | 0.22 (0.10-0.33) | 0.26 (0.12-0.43) | 0.251 |
| IL10 / IL1 | 1.06 (0.56-2.00) | 1.66 (0.89-3.99) | 0.110 |
| IL10 / IL1a | 12.25 (4.76-21.09) | 13.16 (4.92-32.79) | 0.681 |
| IL10 / IL1b | 1.09 (0.55-1.80) | 1.42 (0.83-2.38) | 0.128 |
| **Exhaled breath condensate** | | | |
| IL4 / IL2 | 1.84 (1.46-2.11) | 1.78 (1.60-2.72) | 0.535 |
| IL4 / IL6 | 16.65 (14.13-21.98) | 17.60 (15.67-23.15) | 0.498 |
| IL4 / IL8 | 4.30 (3.20-8.73) | 4.28 (3.00-5.40) | 0.761 |
| IL4 / IFNγ | 8.61 (7.13-10.00) | 7.81 (6.96-9.19) | 0.412 |
| IL4 / TNFα | 2.36 (2.10-2.82) | 2.03 (1.40-2.71) | 0.244 |
| IL4 / IL1 | 2.80 (1.72-14.68) | 12.00 (1.76-32.09) | 0.690 |
| IL4 / IL1a | 15.18 (10.67-21.82) | 20.83 (10.89-39.50) | 0.104 |
| IL4 / IL1b | 2.29 (1.93-3.32) | 2.16 (1.82-2.50) | 0.782 |
| IL10 / IL2 | 0.32 (0.00-0.46) | 0.28 (0.00-0.44) | 0.399 |
| IL10 / IL6 | 2.62 (0.00-3.56) | 0.00 (0.00-3.30) | 0.498 |
| IL10 / IL8 | 0.71 (0.00-1.72) | 0.44 (0.00-0.76) | 0.346 |
| IL10 / IFNγ | 1.46 (0.00-1.67) | 1.42 (0.00-2.31) | 0.941 |
| IL10 / TNFα | 0.40 (0.00-0.48) | 0.16 (0.00-0.43) | 0.467 |
| IL10 / IL1 | 0.39 (0.00-2.14) | 0.41 (0.00-2.63) | 0.900 |
| IL10 / IL1a | 2.27 (0.00-3.64) | 1.68 (0.00-3.36) | 0.413 |
| IL10 / IL1b | 0.42 (0.00-0.71) | 0.37 (0.00-0.58) | 0.671 |

Data presented as median (interquartile range)

IL, interleukin; IFN, interferon; TNF, tumour necrosis factor
